# Temporomandibular joint and Giant Panda’s (Ailuropoda melanoleuca) adaptation to bamboo diet

**DOI:** 10.1038/s41598-021-93808-2

**Published:** 2021-07-09

**Authors:** Pekka K. Vallittu, Juha Varrela, Jukka Salo, Li Rengui, Ling Shanshan, Huang Shan, Hemin Zhang, Pekka Niemelä

**Affiliations:** 1 Department of Biomaterials Science, Institute of Dentistry, University of Turku, Lemminkäisenkatu 2, 20520 Turku, Finland; 2 City of Turku Welfare Division, Turku, Finland; 3 Department of Oral Development and Orthodontics, Institute of Dentistry, University of Turku, Turku, Finland; 4 Biodiversity Unit, University of Turku, Turku, Finland; 5 China Conservation and Research Center for Giant Panda, Dujiangyan, Sichuan China; 6 Key Laboratory of State Forestry and Grassland Administration on Conservation Biology of Rare Animals in the Giant Panda National Park, Dujiangyan, Sichuan China; 7 Qionglai Mountains Conservation Biology of Endangered Wild Animals and Plants National Permanent Scientific Research Base, Dujiangyan, Sichuan China

**Keywords:** Developmental biology, Evolution

## Abstract

Here, we present new evidence that evolutionary adaptation of the Ailuripodinae lineage to bamboo diet has taken place by morphological adaptations in the masticatory system. The giant panda in the wild and in captivity removes without an exception the outer skin of all bamboo shoots, rich in abrasive and toxic compounds, by the highly adapted premolars P3 and P4. The temporomandibular joint (TMJ) allows sidewise movement of the jaw and the premolars can, in a cusp-to-cusp position, remove the poorly digestible outer skin of the bamboo before crushing the bamboo with molars. Based on the evidence presented here, we suggest that adaptation of TMJ to lateral movement for enabling cusp-to-cusp contact of premolars is the crucial evolutionary factor as which we consider the key to understand the Ailuropodinae lineage adaptive pathway to utilize the bamboo resource.

## Introduction

Giant pandas (*Ailuropoda melanoleuca*, Ursidae: Ailuropodinae) have adapted to a herbivorous diet and depend almost exclusively on bamboo (Poaceae: Bambusoidea)^1–3^. The diversion from the omni/carnivorous Ursidae took place during the late Miocene/early Pleistocene and was probably associated with the large bamboo resources available at that time^4,5^. The adaptation process from omni/carnivore to herbivore has resulted in extensive evolution in cranial shape and in the morphology of teeth and dentition^5–7^.

The giant panda appears not to have evolved a gut microbiota compatible with its newly adopted bamboo diet^8^. In addition, the obvious deficient in cellulose- and hemicellulose digesting enzymes as well as carnivore like anatomy of the digestive track have further deepened the ongoing debate around the adaptive pathways of giant panda’s evolution towards a bamboo diet^9–11^.

In the evolutionary adaptation of the giant panda from omni/carnivore to herbivore, mechanical processing of the cellulose-rich food has exerted significant evolutionary pressure on its masticatory function, leading to changes in the morphology of the teeth. A specific feature in panda’s feeding behaviour is its habit to remove the outer layer from the stalk of bamboo before eating and our aim is to analyse how the masticatory system and function has adapted to this feeding pattern.

Compared to the carnivorous polar bear (*Ursus maritimus*), the bamboo-eating giant panda has much larger molar and premolar areas to crush food with^12–14^. Interestingly, premolars P3 and P4 are narrow in width and there is only minor internal (lingual) cusp which suggest that premolars may have specialized function in the mastication. For cutting or grinding function of teeth cusp-to-cusp position of upper and lower teeth by lateral movement of the mandible is typically needed.

Temporomandibular joint (TMJ) has a crucial role in the jaw movement and masticatory function. However, studies on the adaptations of the TMJ on a herbivorous diet are practically missing. Here we present evidence of adaptation of the TMJ from a functional perspective. The anatomical features of the condylar heads of TMJ of giant panda are compared to those of brown bear (*Ursus arctos*) and polar bear (*Ursus maritimus*). We suggest that the anatomy of the condylar heads of the mandible of carnivore or omnivore bears are adapted to a hinge and rotational movement whereas the condylar heads of herbivore bear have features which allow lateral movement of the jaw for removing the poorly digestible and toxic outer skin from the inner layers of the culm of bamboo before eating^15–18^.

## Materials and methods

The condylar heads of the TMJ of six polar bears, six brown bears and two giant pandas were scanned at the Natural History Museum (University of Helsinki, Helsinki, Finland) and at the China Conservation and Research Center for the Giant Panda (CCRCGP, Dujiangyan, China) using an Emerald laser scanner (Planmeca, Helsinki, Finland). The resultant polygonal meshes were saved in STL-format and imported into a CAD application (Rhinoceros version 5, Robert McNeel & Associates, Seattle, WA, USA). The polygonal meshes of each condyle were then manually oriented and scaled to a standardized spatial arrangement for a superimposed visualization of the morphology of the condyles. The mandibles were photographed from the occlusal direction and the frontal direction for demonstrating the dentition and especially the anterior and canine teeth relationship. Using one giant panda skull, the lateral movement of the mandible and guiding path of the incisor—canine region of the dentition was simulated and video recorded. The intercondylar angle was defined from the long axis of the condylar heads from the occlusal view of the lower jaw.

## Results

**Figure 1:**
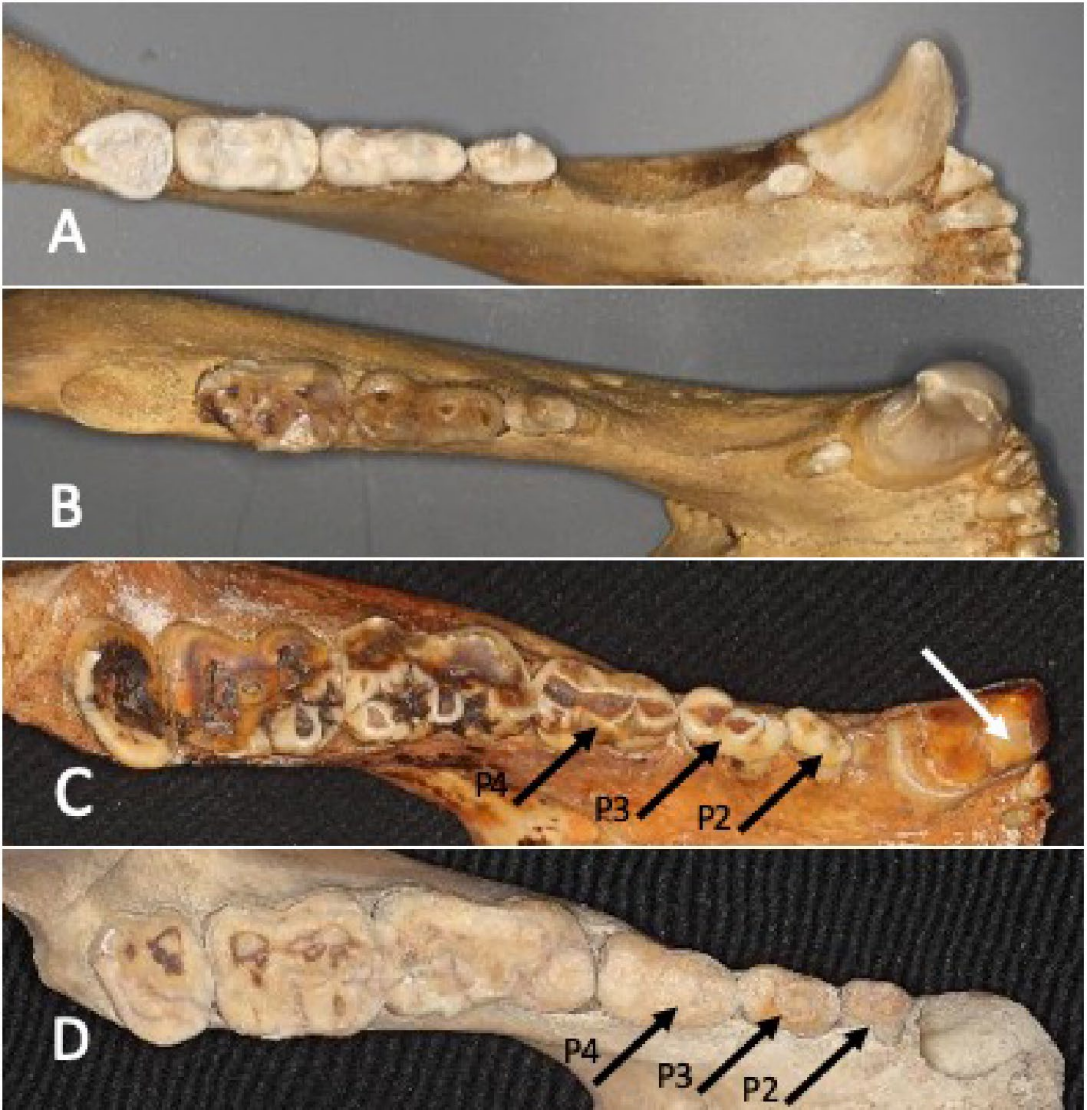
Occlusal view of the dentition showing three incisors, a canine, three premolars, and three molars in the mandible of giant panda. (**A**) Polar bear, (**B**) brown bear, (**C**) giant panda (old) and (**D**) giant panda (young). The white arrow shows the wear facet in the lower canine due to lateral movement of the jaw. Occlusally narrow (black arrows) P3 and P4 can slide the cusp-to-cups cutting position in lateral movement of the mandible.

**Figure 2:**
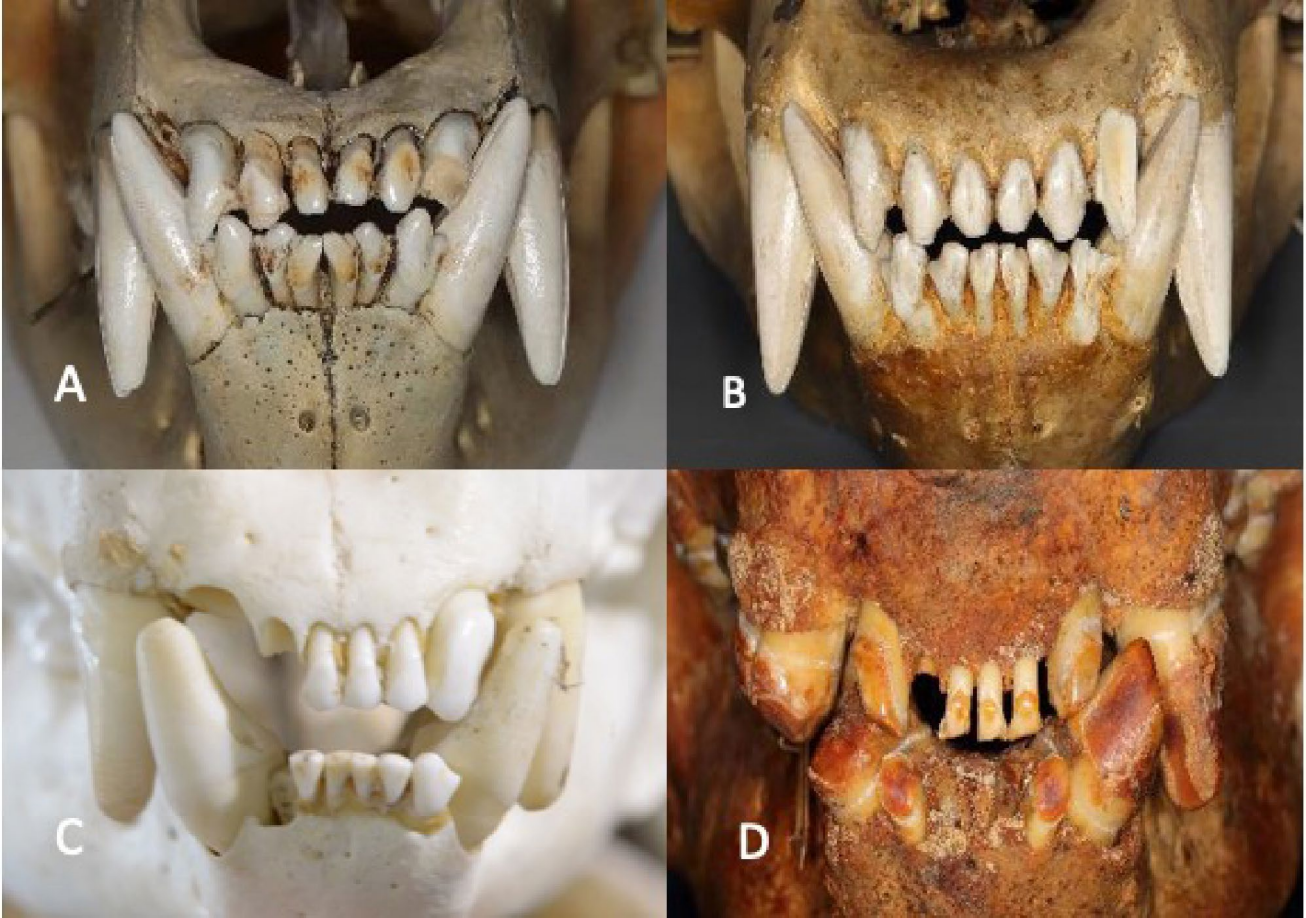
Frontal view of the incisors and canine showed that the third incisor was larger than the other two incisors. Giant panda had the largest third incisors. (**A**) Polar bear, (**B**) brown bear, (**C**) giant panda (young) and (**D**) giant panda (old).

The occlusal view of the dentition showed three incisors, a canine, three premolars, and three molars in the mandible of the giant panda. The polar bear and brown bear both had three incisors, a canine, two premolars and three molars (Fig. 1). The premolars of the giant panda, particularly P4 and P3, were large and mesio-distally elongated differing from those of the polar bear and brown bear. Compared to molars used for crushing the bamboo, P4 and P3 were more conical in form and showed distinct horizontal wear facets in the buccal cusps. The size of the incisors increased from the first to the third in all three species (Fig. 2). However, the size difference between the third and second was largest in the giant panda. Furthermore, the third incisors of the giant panda showed more vertical wear facets resulting from a sliding movement of the mandible.

**Figure 3:**
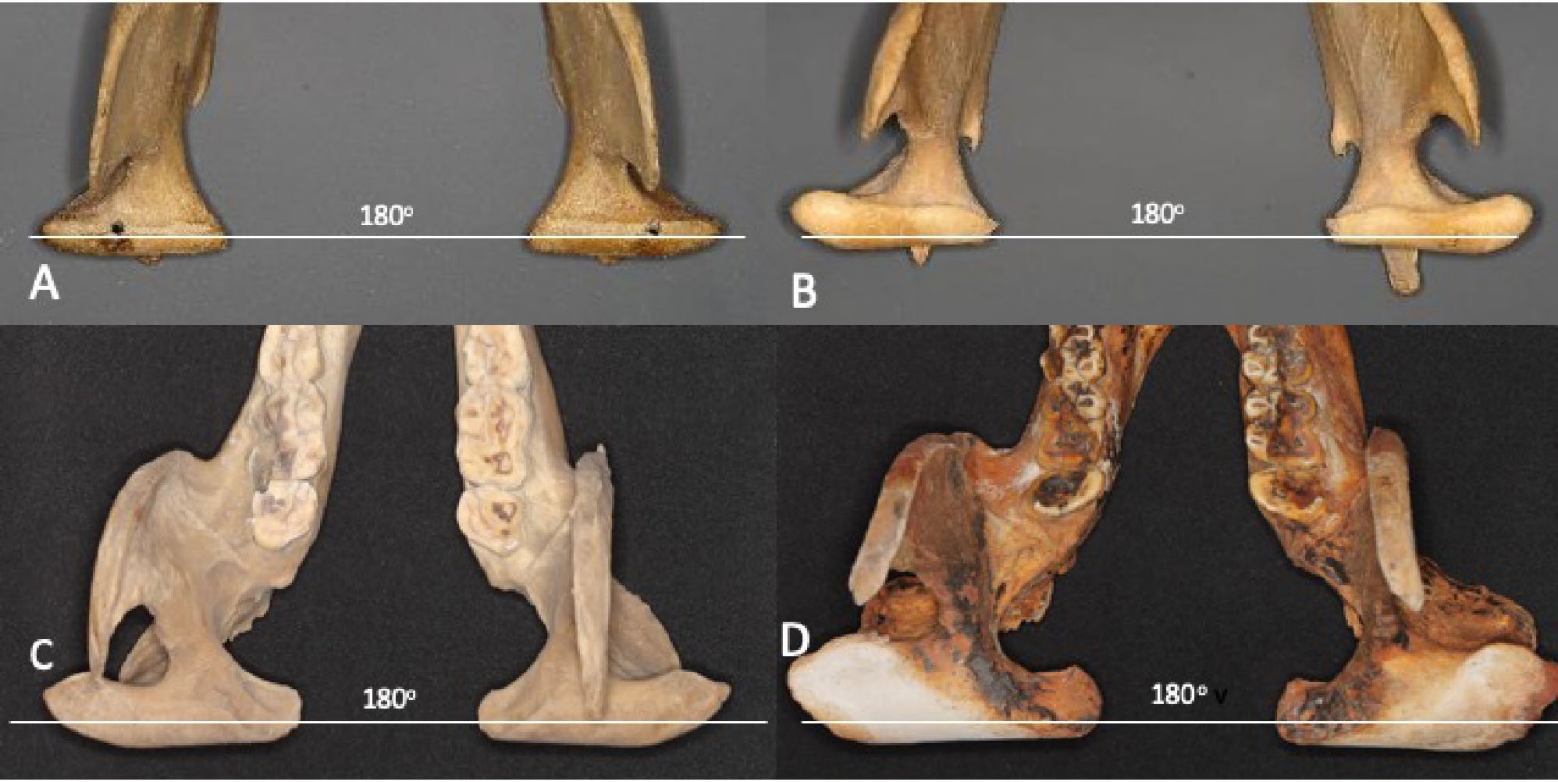
Condylar heads of the giant panda showed a widening of the lateral end with intercondylar angle of 180°. (**A**) Polar bear, (**B**) brown bear, (**C**) giant panda (young) and (**D**) giant panda (old).

**Figure 4:**
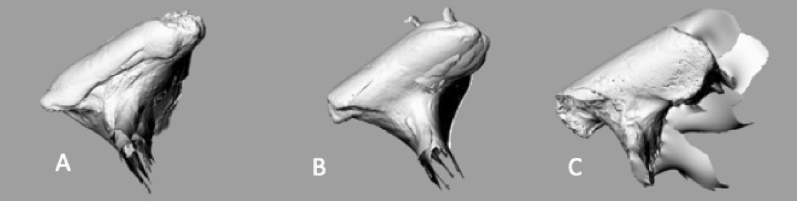
Superimposed laser scans showed the lateral end of the condylar hear of giant panda to be a flat and widened surface. (**A**) Polar bear, (**B**) brown bear, (**C**) giant panda.

In the occlusal view, the condylar heads of the polar bear were cylindrical in shape whereas in the brown bear and giant panda, a widening of the condylar heads was observed with a flat surface at the lateral end (Fig. 3). Laser scans demonstrated that the lateral widening of the condylar head was largest in the giant panda (Fig. 4).

Video simulation of the lateral movement of the mandible of the giant panda showed that the lateral occlusion scheme was controlled by the upper third incisor and lower canine of the mediotrusion side (Movie 1) (Fig. 2). Movie 1 shows the function of the premolars in removing the outer skin of bamboo culm. The role of “panda’s thumb”, giving additional grip around the leaf wad can also be observed, as well as the cutting function of the premolars. The video also shows the obvious olfactory testing of each bamboo plant by panda, a behavior and function which has not yet been explained. Condylar heads of the polar bear, brown bear and giant panda were on the same long axis demonstrating the intercondylar angle to be 180° (Fig. 3). In centric occlusion upper and lower P3 and P4 were not in cusp-to-cusp contact. Our observations show that during the skimming of bamboo, the giant panda uses lateral movement of the mandible to bring the premolars to cusp-to-cusp position which is necessary for removing the outer layer of the culm.

## Discussion

In June 2019, the skulls of two giant pandas were examined from the collection of the CCRCGP. These pandas had lived in the wild (in situ) thereby providing insight into the role of their natural environment in the adaptation of the species to a bamboo diet during evolution. Investigation of these skulls revealed that the masticatory system of the giant panda shows special adaptations in the function and morphology of the mandibular condyle, premolars and incisors that have not been previously described. Previously it has been shown that although the diet of giant pandas is almost exclusively bamboo, the alimentary tract is similar than that of carnivores^1,19,20^. Therefore, it was of interest to investigate anatomical features of the TMJ as a key bony structure for the function of the masticatory system, the beginning part of the alimentary tract, namely the oral cavity. Coevolution between the masticatory system and the digestive system is still an open question.

A special feature in panda’s feeding behaviour, related to eating bamboo, is the habit to skim the outer green skin of the hard bamboo culm. Although the feeding preferences have a seasonal shift, the culm is consumed and skinned during all the year^3^. While eating culm, the outer skin is removed for reasons which until now have remained poorly understood^2,19–21^. To have a firm grip of the bamboo (culm and wad of leaves) during mastication, giant pandas have evolved a pseudo-thumb from the radial sesamoid bone, which is an example of adaptive evolution of an organ to facilitate feeding^22,23^. In addition, the distal premolars, P3 in particular, seem to have evolved morphologically for skimming the culm, distinct from the crushing function of the molars^24^. A cusp-to-cusp position of the premolars is used during skimming and it requires lateral movement of the mandible. The adaptations of the TMJ, especially the condylar head, allows in addition to the basic hinge movement a sliding component that is necessary for the lateral shift of the mandible.

Although the giant panda’s anatomy has been extensively described the features of the mandibular condyles in relation to the jaw movement have not received attention. An insight into the anatomy of mandibular condyles was made by Wanpo who paid attention to the length of the condyles transversally and their potential effect on mechanical efficiency for the masticatory muscles^25,26^. The chewing function of the giant panda seems to have features similar to that seen in herbivorous vertebrates: chewing cycle involves lateral movement. In most herbivores the canines are small or missing with no restricting effect on lateral movement whereas carnivores have typically prominent upper canines which restrict lateral movement of the jaw. In humans, on the other hand, prominent but small canines control lateral movement of the mandible with a neuromuscular sensory feedback system that impacts the muscle activity and mandibular movement^27,28^.

The TMJ of a herbivorous or omnivorous mammal is a complex ginglymoarthroidal joint combining features of both a ginglymus (hinging) and an arthrodial (sliding) joint. This is in contrast to the TMJ of a carnivore, which is of the ginglymus type where the condyle is cylindrical in form allowing hinging and rotational movement (open-close movement). Although the TMJ consists of several anatomical structures such as the glenoid fossa and articular disc, it is the anatomy of the mandibular condyle (*capitulum mandibulae*) that largely modifies the movement of the mandible^27^*.* When food is masticated by herbivores or omnivores, the condylar heads and mandible slide laterally allowing the cusps, fossae, grooves and ridges of premolars and molars to effectively grind the food. The present study demonstrated that the condylar head of the polar bear was cylindrical in shape whereas the condylar heads of brown bear had slightly flattened upper lateral surfaces. This could relate to the differences in diet and biting function of carnivorous and omnivorous mammals. The condylar head of the TMJ of the giant panda had a large flat lateral extension allowing lateral movement of the mandible in the biting cycle.

Interestingly, it was found that the mandibular intercondylar angle of polar bears, brown bears and giant pandas was 180°, which differs from that of e.g. humans (omnivores) with a mandibular intercondylar angle of 140°^28^*.* The intercondylar angle varies among different species depending on the diet and type of masticatory movement^29^. An intercondylar angle less than 180° allows lateral shift (so-called Bennett movement) of the mandible to take place as a component of lateral movement of the lower jaw for more efficient grinding of food^30^. It is possible that the anatomy of the condylar head of giant pandas has evolved to allow limited lateral jaw movements, sufficient for eating bamboo and removing stalk, yet distinct from those seen in most other herbivores.

During the lateral movement of the mandible of the giant panda, the upper third incisor and lower canine slide against each other and guide the movement of the jaw. The guiding teeth are on the mediotrusion side which differs from the lateral occlusal scheme of e.g. of humans where guiding takes place between upper and lower canines on the laterotrusion side. Davies reported that the third incisor of the giant panda is larger than in *Ursus* in general^13^. It seems likely that the larger size of both the crown and root of the third incisors is an adaptation to the lateral force component of the biting cycle. The lateral guiding movement resulted in wear on the lingual surface of the lover canines. The older of the two giant pandas showed severe wear of the lower canine and upper third incisor. Wear patterns were not found in the mediotrusion side canines of the polar bear and brown bear. The large size of the third incisor in the giant panda seems to relate to its guiding role in the lateral movement of the mandible that requires an ability to withstand functional wear. An interesting observation of this study was that the large and robust upper canines that typically have the key role in the lateral occlusal scheme of omnivores, did not have a function in the masticatory cycle of the giant panda. The upper canines of the giant panda project forward more than in *Ursus* which may be associated with its limited role in the masticatory activity in addition to the basic role of canines in fighting. The distal premolars, P3 in particular, are conical and relatively narrow, distinct from the molars which have flat and wide occlusal surfaces. These differences seem to relate to the role of the premolars in the skimming of the bamboo stem in contrast to the crushing function of the molars.

It appears that the function of skimming the outer skin (Movie 2) is either related to elimination of the silica particles in the outer skin of bamboo culm, or to avoid the possible cyanide compounds present in some of the bamboo species. The phytolith silica particles in outer skin and leaves are widespread among the Chinese bamboo species^31–33^, and considered to be adaptation against herbivory. The phytoliths present a major erosive agent to the molars of giant panda, and morphological evolution of the premolars may be the single most important evolutionary factor that made it possible for the panda lineage to adapt to the bamboo-dominated diet.

Cyanogenic glygosides are suggested to be present in many species of bamboos and their role as anti-herbivory agents by bamboo have been discussed^11,31–33^. It is probable that the removal of the outer skin of bamboo culm by the masticatory system may also serve as adaptation to avoid the intake of cyanogenic glucosides, in addition to removing the phytoliths which could cause wear of toughened enamel structure of teeth^34^*.*

We conclude that the masticatory system of giant pandas revealed that specialized morphology of the condylar heads of the TMJ being shaped to for lateral movement of the mandible to remove poorly digestible outer skin of the bamboo culm, and the role of the upper third incisor and lower canine in directing jaw movement are the key characteristics which played an important role in evolutionary adaptation to a bamboo-dominated herbivorous diet.

## Supplementary Information

Supplementary Information 1.

Supplementary Video 1.

Supplementary Video 2.

Supplementary Video 3.

## References

[1] Nie J, Speakma JR, Wu Q, Zhang C, Hu Y, Xia M, Yan L, Hambly C, Wang L, Wei W, Zhang M, Wei F (2015). Exceptionally low daily energy expenditure in the bamboo-eating giant panda. Science.

[2] Schaller GB, Jinchu H, Wenshi P, Jing Z (1985). The Giant Pandas of Wolong.

[3] Long Y, Lü Z, Wang D, Zhu X, Wang H, Zhang Y, Pan W, Lindburg D, Baragona K (2004). Nutritional strategy of Giant Pandas in the Qingling Mountainsa of China. Giant Pandas—Biology and Conservation.

[4] Gittleman JL (1999). Hanging bears from phylogenetic trees: Investigating patterns of macroevolution. Ursus.

[5] Jin C, Ciochon RL, Dong W, Hunt RM, Jinyi Liu K, Jaeger M, Zhu Q (2007). The first skull of the earliest giant panda. Proc. Natl. Acad. Sci..

[6] Figueirido B, Palmqvist P, Pérez-Claros JA, Dong W (2011). Cranial shape transformation in the evolution of the giant panda (Ailuropoda melanoleuca). Naturwissenschaften.

[7] Zhang S, Pan R, Li M, Oxnard C, Wei F (2007). Mandible of the giant panda (Ailuropoda melanoleuca) compared with other Chinese carnivores: Functional adaptation. Biol. J. Lin. Soc..

[8] Xue Z, Zhang W, Wang L, Hou R, Zhang M, Fei L, Zhang X, Huang H, Bridgewater LC, Jiang Y, Jiang C, Zhao L, Pang X, Zhang Z (2015). The bamboo-eating giant panda harbors a carnivore-like gut microbiota, with ecessive seasonal variations. MBio.

[9] Guo W, Mishra S, Zhao J, Tang J, Zeng B, Kong F, Ning R, Li M, Zhang H, Zeng Y, Tian Y, Zhong Y, Luo H, Liu Y, Yang J, Yang M, Zhang M, Li Y, Ni Q, Li C, Wang C, Li D, Zhang H, Zuo Z, Li Y (2018). Metagenomic study suggests that yje gut microbiotra of the giant pands (Ailuropoda melanoleuca) may not be specialized for fiber fermentation. Front. Microbiol..

[10] Zhu L, Yang Z, Yao R, Xu L, Chen H, Gu X, Wu T, Yang X (2018). Potential mechanism of detoxification of cyanide compounds by gut microbiomes of bamboo-eating pandas. mSphere.

[11] Zhu L, Wu Q, Dai J, Zhang S, Wei F (2011). Evidence of cellulose metabolism by the giant panda gut microbiome. PNAS.

[12] Ungar PS (2015). Mammalian dental function and wear. A review. Biosurf. Biotribol..

[13] Davis DD (1964). The giant panda. A morphological study of evolutionary mechanisms. Fieldiana Zool. Mem..

[14] Sacco TV, Valkenburgh B (2004). Ecomorphological indicators of feeding behaviours in the bears (Carnivora: Ursidae). J. Zool..

[15] Liese W (2002). The Anatomy of Bamboo Culms.

[16] Tarou LR, Williams J, Powell DM, Tabet R, Allen M (2005). Behavioral preferences for bamboo diet in a pair of captive giant pandas (*Ailuropoda melanoleuca*). Zoo Biol..

[17] Abduo JM (2015). Tennant, Impact of lateral occlusion schemes: A systematic review. J. Prosthet. Dent..

[18] Avivi-Arber L, Sessle BJ (2017). Jaw sensimotor control in healthy adults and effectsof ageing. J. Oral. Rehabil..

[19] Wei F, Hu Y, Yan L, Noe Y, Wu Q, Zhang Z (2014). Giant pandas are not an evolutionary cul-de-sac: Evidence from multidisciplinary research. Mol. Biol. Evol..

[20] Dierenfeld, E. S. Chemical Composition of Bamboo in Relation to Giant Panda Nutrition. In *The bamboos*, 205–211 (Academic Press, 1997).

[21] Dierenfeld ES, Hintz HF, Robertson JB, Van Soest PJ, Oftedal OT (1982). Utililization of bamboo by giant panda. J. Nutr..

[22] Endo H, Yamagiwa D, Hayashi Y, Koie H, Yamaya Y, Kimura J (1999). Role of the giant panda´s ´pseudo-thumb´. Nature.

[23] Salesa MJ, Anton M, Peigne S, Morales J (2006). Evidence of a false thumb in a fossil carnivore clarifies the evolution of pandas. Proc. Natl. Acad. Sci. U S A..

[24] Huang W (1993). The skull, mandible and dentition of Giant Pandas (Ailuropoda) Morphological characters and their evolutionary implications. Vertebrata PalAsiatica.

[25] Wanpo H (1993). The skull, mandible and dentition of giant pandas (ailuropoda): Morphological characters and their evolutionary implications. Vertebrata PalAsiatica.

[26] Gidmark NJ, Tarrant JC, Brainerd EL (2014). Convergence in morphology and masticatory function between the pharyngeal jaws of grass carp, *Ctenopharyngodon idella*, and oral jaws of amniote herbivores. Biologist.

[27] Ramfjord, S., Ash, M. M. Functional anatomy of the temporomandibular joint. In *Occlusion*, edn 3, 9–18 (WB Saunders, 1983.

[28] Eisenburger M, Haibitz B, Schmelzeisen R, Wolter S, Tschernitschek H (1999). The human mandibular intercondylar angle measured by computed tomography. Arch. Oral. Biol..

[29] Schumacher GH (1961). Funktionell Morphologie der Kaumuskulatur.

[30] Fanucci E, Spera E, Ottria L, Barlattani A, Fusco N, Mylonakou I, Broccoli P, Barlattani A, Simonetti G (2008). Bennet movement of mandible: A comparison between traditional methods and a 64-slices CT scanner. Oral. Impl..

[31] Parsons, J. L. Bamboo nutritional composition, biomass production, and palatability to giant pandas: disturbance and temporal effects. Thesis, Mississippi State University, UMI Number 3590228 (2013).

[32] Gu Y, Liu H, Wang R, Yu J (2016). Phytoliths as a method of identification for three genera of woody bamboos (Bambusoideae) in tropical southwest China. J. Archaeol. Sci..

[33] Yin X, Xu Y, Lin T, Liang Q, Yang B, Duan C (2016). Silicon morphology in bamboo. BioResources.

[34] Weng ZY, Liu ZQ, Ritchie RO, Jiao D, Li DS, Wu HL, Deng LH, Zhang ZF (2016). Giant panda's tooth enamel: Structure, mechanical behavior and toughening mechanisms under indentation. J. Mech. Behav. Biomed. Mater..

