# Supplementary material for: Temporomandibular joint and Giant Panda’s (Ailuropoda melanoleuca) adaptation to bamboo diet
Source: Sci Rep. 2021 Jul 9;11:14252. doi: 10.1038/s41598-021-93808-2 (PMC8271028; doi:10.1038/s41598-021-93808-2)
Supplement: Supplementary file 1 — Supplementary Information 1. [file 41598_2021_93808_MOESM1_ESM.docx]

Supplementary Movie 1:

Video simulation of the lateral movement of the mandible of giant panda showed that the lateral occlusion scheme was controlled by the upper third incisor and lower canine of the mediotrusion side and that the condylar head allows lateral movement of the jaw. Lateral movement is used for getting premolars to cusp-to-cusp positions when skimming poorly digestible outer skin of the bamboo culm.

Supplementary Movie 2:

Skimming the outer skin of bamboo is typical for giant panda which relate to elimination of the high silica particles in the outer skin of bamboo, or to avoid the possible cyanide compounds present in some of the bamboo species. The video shows the function of the premolars in removing the outer skin of bamboo culm, here the bamboo *Phyllostachys* sp. The role of “panda’s thumb”, giving additional grip around the leaf wad can also be observed, as well as the cutting function of the premolars. The video also shows the obvious olfactory testing of each bamboo plant by panda, a behavior and function which has not yet been explained. Panda is 金宝宝 (*Jīn Bǎobǎo*), female (6 years old), China Giant Panda Studbook number 941 (CCRCGP).

Supplementary Movie 3:

Video shows lateral movement of lower jaw when removing the outer skin of bamboo culm. Panda is 华豹 *Huá Bào*, male (7 years old) China Giant Panda Studbook number 867 (CCRCGP).
